# Undergraduate exposure and confidence to amputations and amputee care: a national survey of final-year UK medical students

**DOI:** 10.1186/s12909-025-08027-4

**Published:** 2025-11-27

**Authors:** Ameen Mahmood, Helena Macdonald, Iihan Ali, Gwennan Shewring, Sofia Breeze, Matthew Wordsworth, Amputation Exposure Collaborative, Aashlesha Galla, Abinaya Arulalagan, Aditya Bose-Mandal, Agata Baczynska, Agnese Tiranti, Alice Gait, Anas Mohamed, Andrei Balan, Aparajith Sathish Kumar, Becky Leveridge, Bethany Hatten, Charlotte Cross, Charlotte Nicollin, Charlotte Rose Bairstow, Chandan Sekhon, Emily Taylor, Gurjivan Sohal, Hiya Grover, Ivan Abbott, Jemi Maliyil, Jenin Ola, Judy Elfakharany, Kai Jian Chin, Kojo Dankwa, Lavanya Natarajan, Leo Hird, Lucy Bowden, Malaika Ali, Mark Ramzy-Riad, Mikesh Patel, Nishka Patel, Omar Farooq, Rananjay Singh, Ranoo Rebaz Jamal, Roni Parnes, Roshni Patel, Ryan Baguley, Saleha Irshad, Samuel Gamblin, Sameera Oruganti, Shivam Kotecha, Simran Khutan, Tom Renny, Vaishvi Dalal, Vanessa Evans, Wibah Ibrahim Adnan, Az-Zubayr Sahraoui

**Affiliations:** 1 School of Medicine, Imperial College London, London, UK; 2 School of Medicine, King’s College London, London, UK; 3 School of Medicine, University of Liverpool, Liverpool, UK; 4 Norwich Medical School, University of East Anglia, Norwich, UK; 5 Department of Surgery, Imperial College Healthcare NHS Trust, London, UK

**Keywords:** Amputations, Amputee care, Undergraduate curriculum, Medical education, Collaborative research

## Abstract

**Background:**

Amputations are complex procedures with profound physical, psychological, and social impacts. Despite their clinical importance, amputation care is poorly represented in UK undergraduate medical curricula. This study evaluates final-year medical students’ exposure, confidence, and perceptions regarding amputation care.

**Methods:**

A national, cross-sectional survey was distributed to final-year students across all 40 UK medical schools between August and December 2024. The survey assessed formal teaching, clinical exposure, confidence across key domains, and perceived barriers. Statistical analysis included Mann–Whitney U and Kruskal–Wallis tests.

**Results:**

Of 654 respondents, 70.9% reported no formal teaching on amputation care, and 18.0% had not encountered an amputation patient. Only 11.5% rated existing teaching as effective. Confidence was low regarding the psychological (43%) and physiological (51%) management of patients with amputions, and 71.9% lacked familiarity with support services. Students with teaching or clinical exposure reported significantly higher confidence levels (p < 0.001). Major barriers identified included limited teaching (79.4%) and inadequate clinical exposure (61.5%). Encouragingly, 71.4% expressed willingness to attend future workshops.

**Conclusions:**

Significant gaps exist in undergraduate education on amputation care, contributing to low student confidence. Structured teaching, enhanced clinical exposure, and collaboration with specialist organisations are urgently needed. A national framework would ensure consistent preparation for managing patients with amputations across UK graduates.

**Supplementary Information:**

The online version contains supplementary material available at 10.1186/s12909-025-08027-4.

## Background

Amputation is a major surgical intervention with far-reaching physical, psychological, and societal impacts [1]. In the UK, approximately 5,000 major limb amputations are performed annually [1], with each procedure costing the NHS an estimated £65,000 [2]. Patients undergoing lower limb amputations often experience reduced mobility, which heavily impacts their quality of life and ability to perform activities of daily living [3]. Additionally, the societal costs include loss of productivity, long-term disability support, and substantial rehabilitation needs [4].

Globally, amputations have gained heightened relevance in conflict zones, where the prevalence of traumatic amputations has reached unprecedented levels [5, 6]. In Ukraine alone, it is estimated that since 2022 more than 50,000 military personnel have undergone amputations due to the ongoing war [7, 8]. By comparison, 265 UK service personnel sustained 416 amputations in Afghanistan collectively over a decade [9, 10]. In addition, due to the war in Gaza, it now has the highest number of children with amputations per capita in the world [6, 11, 12]. These figures underscore the growing demand for clinicians equipped to manage complex cases of limb loss and rehabilitation.

Amputation care extends beyond surgery, requiring multidisciplinary management that encompasses pre-operative care, rehabilitation, prosthetic fitting, and psychological support [1]. The collaboration of these specialties is essential for optimising outcomes and improving patients' quality of life [13]. Given the multidisciplinary nature of amputation care, it is likely that most doctors will encounter patients with limb loss during their careers, regardless of specialty. It is, therefore, imperative that medical students are equipped with a fundamental understanding of the conditions leading to amputation and the holistic management required for these patients.

The Royal College of Surgeons of England (RCSEng) explicitly mandates that all medical students should be taught the indications, types and operations, complications, and rehabilitation principles of limb amputation as part of the National Undergraduate Curriculum in Surgery [14]. Studies suggest that when medical students receive limited formal teaching and clinical exposure this contributes to low confidence and preparedness in managing such patients [15, 16].

This study aims to assess the current state of amputation care education among final-year medical students across the UK, focusing on teaching methods, clinical exposure, and perceived barriers to learning. By identifying these gaps, this research seeks to inform future educational interventions to improve knowledge, confidence, and preparedness in managing patients with amputations.

## Methods

### Study design and ethical approval

This study employed a national, multi-centre, cross-sectional survey design to explore final-year medical students' perceptions of knowledge, and experiences with amputations and amputee care, including teaching, clinical exposure, and multidisciplinary management. Ethical approval was obtained from the Imperial College Education Ethics Review Process (EERP2324-107), ensuring compliance with the Declaration of Helsinki and GDPR regulations. Participation was voluntary and anonymous, with implied consent provided when participants voluntarily completed the survey after reading the information sheet. Inclusion criteria included all final-year medical students across UK medical schools. Exclusion criteria included non–final-year students and students not enrolled at a UK medical school.

### Survey development and distribution

A bespoke questionnaire using Likert-scale items was developed by the co-authors, following a review of the literature and consultation with experts in amputation care and medical education. To ensure content validity, the draft survey was shared with Blesma [17] (a non-profit organisation supporting limbless veterans) and a consultant who specialises in amputations for feedback. The final survey consisted of 20 questions across five domains (see Additional file 1 for the full survey instrument):

1. **Demographics**: Medical school affiliation and year of study.
2. **Exposure to Amputation Teaching and Care**: Number and types of teaching sessions attended, clinical and hands-on exposure, and perceived barriers to learning.
3. **Confidence Levels**: Knowledge of psychological, physiological, and multidisciplinary aspects of amputation care.
4. **Perceptions and Attitudes**: Views on individuals with amputations and the significance of amputations compared to other surgical procedures.
5. **Feedback on Teaching**: Perceptions of competence and the adequacy of undergraduate training in amputation care.

The survey was hosted on Google Forms and distributed online. Collaborators from all 40 UK medical schools with final-year cohorts recognised by the General Medical Council (GMC) [18] were recruited through PRASSA (Plastic, Reconstructive, and Aesthetic Surgery Student Association) to facilitate dissemination. These collaborators circulated the survey via social media, email campaigns, and medical school networks, maximising national participation and ensuring a representative sample. Data collection commenced on 27 August 2024 and concluded on 28 December 2024.

### Sample size and recruitment

The study targeted an estimated population of 9500 final-year medical students across the UK in 2024 [19]. Using the Raosoft® sample size calculator [20], a sample size of 621 respondents was calculated to achieve 99% confidence with a 5% margin of error. A non-random sampling method was employed to recruit participants from all eligible UK medical schools. To our knowledge, this is the only study in this field.

### Data analysis

Survey responses were handled in Microsoft Excel Version 16.91 and R Version 2025.05.0 + 496 [21] was used for statistical analysis. Descriptive statistics summarised demographic data and survey responses. Ordinal categorical responses were converted into midpoint numeric values to allow for statistical analysis. Data analysis confirmed Cronbach's alpha of 0.8 for the Likert-scale items, indicating good internal consistency. Normality of continuous variables was assessed using the Kolmogorov–Smirnov test. Depending on distribution, comparisons between two groups were performed using either the Mann–Whitney U or independent t-tests. For comparisons involving more than two groups, the Kruskal–Wallis or ANOVA tests were used. Where the Kruskal–Wallis test indicated statistical significance, post hoc pairwise comparisons were performed using Dunn’s test with Bonferroni correction to account for multiple testing. Statistical significance was set at *p* < 0.05.

## Results

### Demographics

**Fig. 1:**
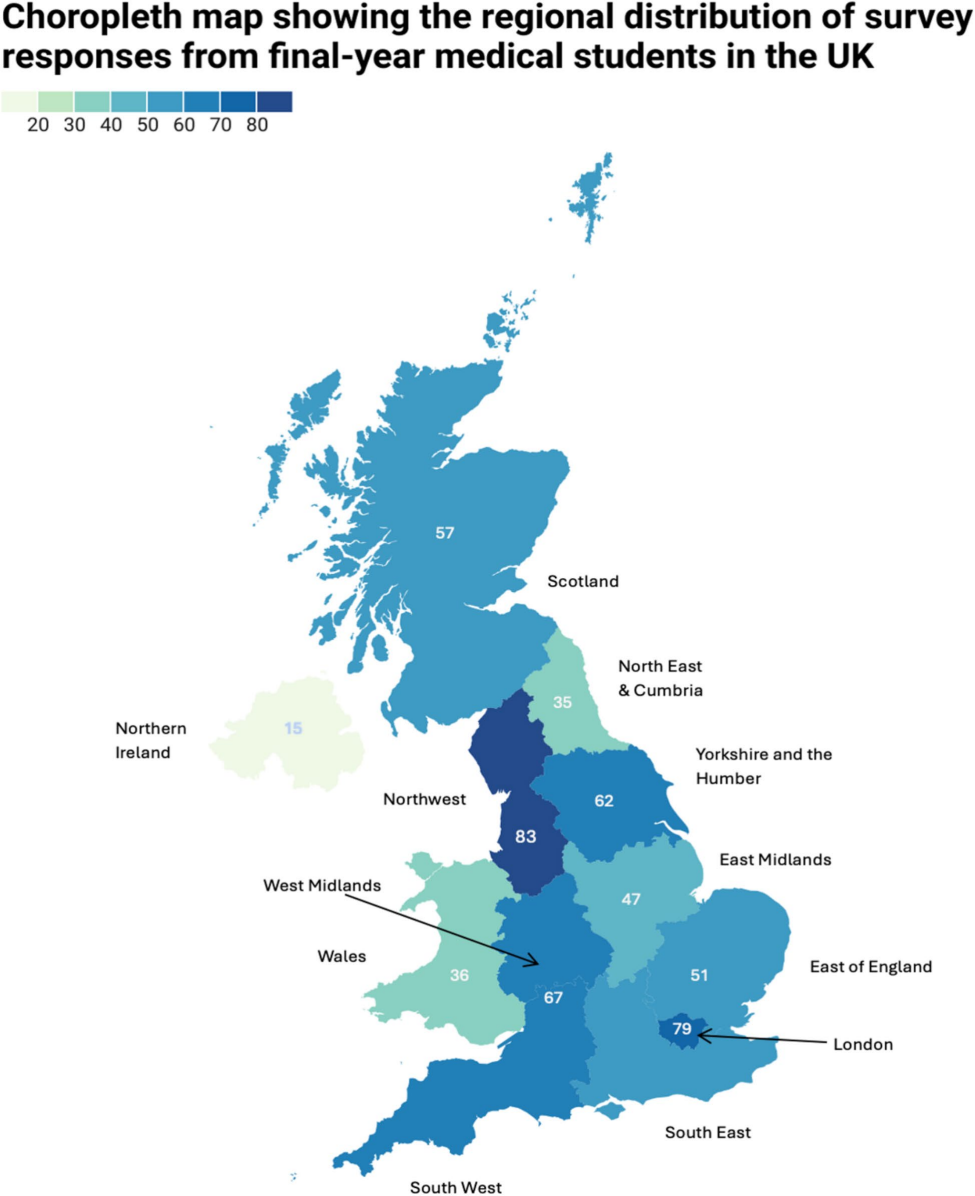
Choropleth map showing the regional distribution of survey responses from final year medical students in the United Kingdom

A total of 654 final-year medical students from all 40 UK medical schools with final-year cohorts participated in the survey. The number of responses varied across institutions, with Liverpool (34 responses), Exeter (29 responses), and Bristol (25 responses) being among the most represented schools. The regional distribution of responses is summarised in Fig. 1.

### Exposure to amputation teaching

Most respondents, 70.9% (*n* = 464) reported no dedicated teaching sessions on amputations and/or amputation care during their medical training. Only 15.4% (*n* = 101) reported one session, while fewer students (13.7%, *n* = 89) reported 2–7 sessions. No respondents had attended more than seven sessions. The national median number of dedicated amputation teaching sessions received was 0 (IQR = 1). National-level data on exposure types is shown in Table 1.

**Table 1:** Total national data of final-year medical students’ exposure to amputations/amputation care within formal undergraduate medical education

	Response	% (n)
Dedicated teaching sessions on amputations/amputation care	None	70.9 (464)
	1	15.2 (101)
	2–3	10.6 (69)
	4–5	2.6 (17)
	6–7	0.5 (3)
	> 7	0 (0)
Formats of teaching on amputations/amputation care experienced	Clinical Observation	20.6 (135)
	Formal Clinical Rotation	18.7 (122)
	Lectures	18.3 (120)
	Small group tutorials/seminars	12.1 (79)
	Clinical Skills	4.0 (26)
	Simulation-Based Training	1.7 (11)
	Other	1.7 (11)
Patients with amputations encountered	None	18.0 (118)
	1–5	55.0 (360)
	6–10	15.3 (100)
	11–15	6.7 (44)
	16–20	1.8 (12)
	> 20	3.1 (20)
Observation of an amputation operation	Yes	23.2 (152)
	No	74.8 (489)
Hands-on experience with procedures directly related to amputation care	Yes	18.0 (118)
	No	78.4 (513)

**Fig. 2:**
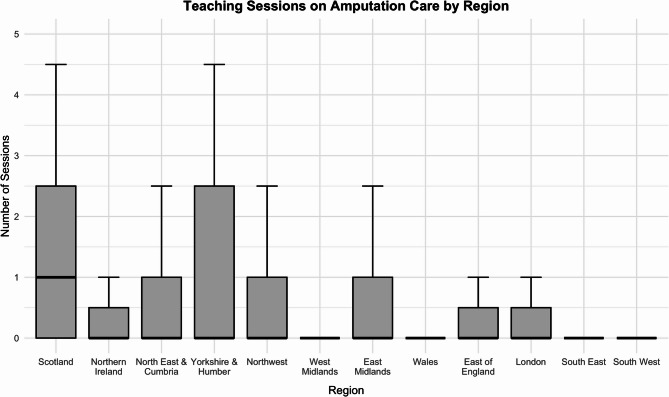
Teaching Sessions on Amputation Care by Region. Boxplot showing the number of dedicated undergraduate teaching sessions on amputations and amputation care reported by final-year medical students across UK regions. The boxes represent the interquartile range (IQR), the horizontal lines indicate medians, and whiskers denote the full range within 1.5 × IQR

Regional analysis revealed significant differences in the number of dedicated amputation teaching sessions received across the UK (*p* < 0.001) (Fig. 2). The median number of teaching sessions reported in Scotland was 1 (IQR = 2.5), compared to a median of 0 sessions in most other regions. Students in Scotland reported significantly greater exposure than those in London, South East, South West, Wales, West Midlands, East Midlands, North East & Cumbria, Northwest, and East of England (adjusted *p* < 0.05). Detailed regional data is provided in Additional file 2.

Regarding teaching formats, clinical observation (20.6%, *n* = 135) and formal clinical rotations (18.7%, *n* = 122) were the most reported teaching formats, followed by lectures (18.3%, *n* = 120) and small group tutorials or seminars (12.1%, *n* = 79). Simulation-based training was uncommon, with only 1.7% (*n* = 11) of students reporting exposure. Students reporting informal learning opportunities, such as student-selected components (SSCs) or recalling attendance at amputation-related conferences, were rare (0.2%).

**Fig. 3:**
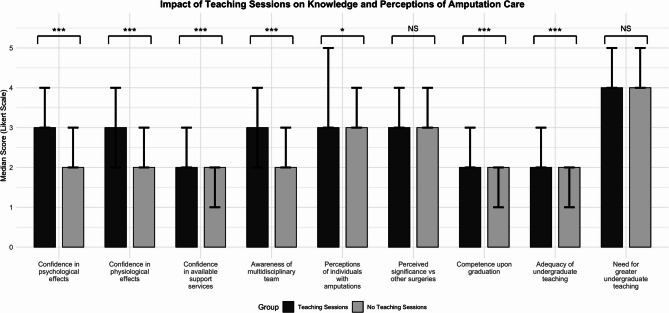
Teaching vs No Teaching Sessions. Bar charts showing the difference in participants’ knowledge and perceptions of amputation care, stratified by whether they had teaching sessions or no teaching sessions. Mann–Whitney U tests were performed to compare median differences, with ns = *p* > 0.05, * = *p* ≤ 0.05, ** = *p* ≤ 0.01, *** = *p* ≤ 0.001. Error bars represent the interquartile range (IQR)

Attending at least one teaching session was significantly associated with higher self-reported confidence across multiple domains of amputation care compared to no teaching sessions (p < 0.001 for psychological, physiological impacts and awareness of multidisciplinary team (MDTs)) (Fig. 3). Detailed comparative statistics between students with and without amputation teaching are presented in Additional file 3.

### Perceived effectiveness of teaching sessions

Among those who had attended teaching sessions only 11.5% (*n* = 75) found the teaching effective or very effective, while 20.7% (*n* = 136) rated it as ineffective or very ineffective. Furthermore, 83.3% (*n* = 545) disagreed or strongly disagreed that their undergraduate education on amputation care had been adequate.

### Clinical exposure to amputation care

Most respondents had limited direct exposure to patients with amputations during medical training. Over half, 55% (n = 360) encountered 1–5 patients, while 18% (n = 118) reported no exposure. Only 21.8% (*n* = 141) had interacted with more than five patients. The national median number of patients with amputations encountered during training was 3 (IQR = 5).

**Fig. 4:**
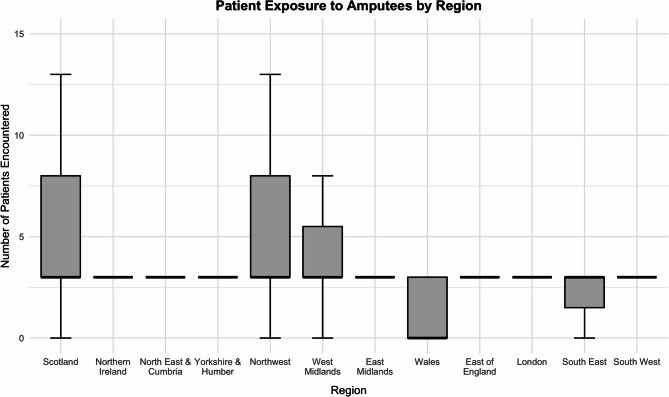
Patient Exposure to Amputees by Region. Boxplot showing the number of patients with amputations encountered during undergraduate medical training reported by final-year medical students across UK regions. The boxes represent the interquartile range (IQR), the horizontal lines indicate medians, and whiskers denote the full range within 1.5 × IQR

Regionally across the UK there was a significant difference in the number of patients with amputations encountered (p < 0.001) (Fig. 4). The median number of patients with amputations encountered in Wales was 0 (IQR = 3), compared to a median of 3 patients in all other regions (adjusted *p* < 0.05). Detailed regional data is provided in Additional file 2.

Regarding procedural exposure, 74.8% (*n* = 489) had not observed an amputation operation, and 78.4% (*n* = 513) had no hands-on experience with procedures related to amputation care, such as wound dressing or stump care.

**Fig. 5:**
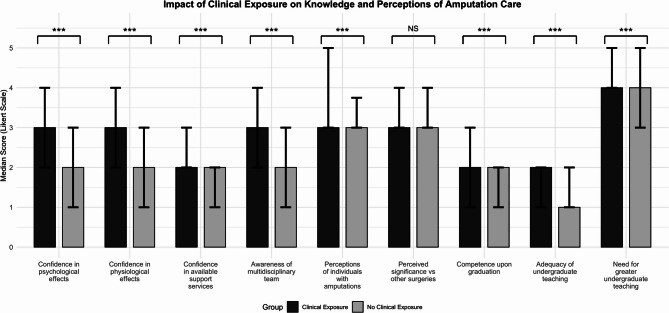
Clinical vs No Clinical Exposure. Bar charts showing the difference in participants’ knowledge and perceptions of amputation care, stratified by whether they had clinical exposure or no clinical exposure. Mann–Whitney U tests were performed to compare median differences, with ns = *p* > 0.05, * = *p* ≤ 0.05, ** = *p* ≤ 0.01, *** = *p* ≤ 0.001. Error bars represent the interquartile range (IQR)

Exposure to one or more patients with amputations significantly improved perceived confidence in knowledge of amputations/amputation care compared to no exposure (*p* < 0.001 for psychological, physiological impacts and awareness of MDTs (Fig. 5). Detailed comparative statistics between students with and without clinical exposure are presented in Additional file 4. Students who had observed an amputation operation demonstrated significantly greater confidence in their knowledge of available resources and services to support patients with amputations, including rehabilitation, prosthetics, and support groups, compared to those without such exposure (*p* = 0.033).

When asked about the perceived significance of amputations relative to other major surgical procedures, a majority (56.7%, *n* = 371) considered them equally significant, while (27.7%, *n* = 181) perceived amputations as more significant.

Notably, perceptions of amputations also were influenced by educational exposure. Among students with some form of teaching or clinical experience, views toward individuals with amputations tended to be more positive (*p* < 0.01). Overall, the majority of respondents held neutral (55.2%, *n* = 361) or positive views (17.1%, *n* = 112 somewhat positive; 23.7%, *n* = 155 very positive).

### Perceived adequacy of clinical exposure

When asked to rate the adequacy of their clinical exposure, 71.4% (*n* = 467) of respondents described it as inadequate or somewhat inadequate. Only 12.1% (*n* = 79) perceived their exposure as adequate, with 0.8% (*n* = 5) indicating more than adequate exposure.

### Confidence in amputation care

Students' confidence in managing patients with amputations was low across key domains. Regarding psychological impacts, 43% (*n* = 281) of students either disagreed or strongly disagreed with feeling confident in their knowledge of the psychological effects that amputations can have on patients. Similarly, 51% (*n* = 334) expressed low confidence in addressing the physiological impacts, and 71.9% (*n* = 470) lacked confidence in identifying and accessing the resources and services available to support patients with amputations. When asked about their awareness of the MDT involved in amputation care, 47.8% (n = 313) of students either disagreed or strongly disagreed with having sufficient awareness. Overall, when asked to assess their overall competence in providing care for patients with amputations upon graduation, 73.7% (*n* = 582) of students reported low confidence. Detailed response breakdowns are presented in Table 2.

**Table 2:** Responses to questions assessing confidence, perceptions, teaching effectiveness, and competence in amputation care among final-year medical students

	Strongly disagree (% (n))	Disagree (% (n))	Neutral (% (n))	Agree (% (n))	Strongly agree (% (n))
I feel confident in my knowledge of the psychological effects that amputations can have on patients	11.8 (77)	31.1 (204)	28.9 (189)	26.0 (170)	2.1 (14)
I feel confident in my knowledge of the impact that amputations can have on a patient's overall physiological health	14.5 (95)	36.5 (239)	25.2 (165)	21.4 (140)	2.3 (15)
I feel confident in my knowledge of the resources and services available to support patients with amputations (e.g., rehabilitation, prosthetics, support groups)	26.7 (175)	45.0 (295)	17.4 (114)	9.2 (60)	1.5 (10)
How much do you agree with the following statement: "I am aware of the various specialists (medical/surgical and allied professionals) involved in the multidisciplinary team for amputation care"?	14.2 (93)	33.6 (220)	23.5 (154)	24.1 (158)	4.4 (29)
How much do you agree with the following statement: "I have received adequate teaching in amputations and/or amputation care during my undergraduate medical education thus far?"	38.6 (253)	44.6 (292)	10.2 (67)	5.0 (33)	1.4 (9)
How much do you agree with the following statement: "Greater exposure and teaching are required on amputations and/or amputation care at the undergraduate level"?	1.8 (12)	2.6 (17)	11.8 (77)	48.2 (316)	35.4 (232)
	Very negatively (% (n))	Somewhat Negatively (% (n))	Neutral (% (n))	Somewhat Positively (% (n))	Very Positively (% (n))
How do you perceive individuals with amputations?	0.3 (2)	3.7 (24)	55.1 (361)	17.1 (112)	23.7 (155)
	Much less significant	Less significant	Equally significant	More significant	Much more significant
How significant do you perceive amputations to be compared to other major surgical procedures (e.g., cardiac surgery, organ transplantation)?	0.5 (3)	6.9 (45)	56.6 (371)	27.6 (181)	8.2 (54)
	Not confident at all (% (n))	Not very confident (% (n))	Neutral (% (n))	Moderately confident (% (n))	Very confident (% (n))
How do you perceive your overall competence in providing care for patients with amputations upon graduation from medical school?	27.8 (182)	45.8 (300)	16.3 (107)	9.3 (61)	0.6 (4)
	No (% (n))	Maybe (% (n))	Yes (% (n))		
Would you attend a teaching session or workshop related to amputations and amputation care?	1.8 (12)	26.7 (175)	71.3 (467)		

### Barriers to learning

Limited teaching sessions (79.4%, *n* = 519) and inadequate clinical exposure (61.5%, *n* = 402) were the most frequently cited barriers to effective learning. Time constraints within the curriculum were also a significant factor (56.3%, *n* = 368), alongside insufficient emphasis on amputation care within assessments (61.3%, *n* = 401).

### Willingness to learn

Despite these deficits, 71.4% (*n* = 467) of respondents indicated that they would attend workshops or sessions dedicated to amputation care if offered. Additionally, 83.8% (*n* = 548) agreed or strongly agreed that greater exposure and teaching are needed at the undergraduate level.

## Discussion

Although the RCSEng mandates that all medical students be taught core competencies in amputation care—including indications, operative techniques, complications, and rehabilitation [14], current undergraduate training falls short of these expectations—70.9% of final-year medical students reported receiving no dedicated teaching sessions on the topic. Among those who had received teaching, only 11.5% rated the sessions as effective or very effective. Clinical exposure was similarly inadequate, 18.0% had not encountered an amputation patient, 74.8% of students had never observed an amputation and 78.4% lacked any hands-on experience in related care. This lack of structured education is concerning given that 5,000 to 6,000 major amputations occur annually in the UK [1], making it highly likely that newly qualified doctors will encounter patients with amputations early in their careers. This discrepancy may reflect both structural factors and recall bias. Amputations may not be included in standard undergraduate clinical rotations, thereby limiting opportunities for observation. Additionally, students may encounter patients with limb loss incidentally during vascular, diabetes, oncology, or rehabilitation placements but not perceive these experiences as ‘amputation teaching,’ leading to under-reporting. These educational deficits are mirrored by consistently low student confidence across key areas of amputation care. A significant proportion of respondents felt unprepared to manage the psychological (43%) and physiological (51%) impacts of limb loss, while 71.9% reported being unfamiliar with the support services available to patients with amputations.

The data indicates that current clinical and educational exposure is insufficient to prepare medical students for the realities they will face upon graduation. The most frequently reported barriers to learning included limited teaching sessions (79.4%), inadequate clinical exposure (61.5%), and insufficient emphasis on amputation care in assessments (61.3%). Notably, 71.4% of students expressed interest in further training, indicating that institutional and structural barriers, not lack of student engagement, are the main obstacles to integrating amputation care into the curriculum.

Although postgraduate training allows for further consolidation and specialist development, it is essential that exposure to amputation care occurs at the undergraduate level to ensure consistent preparation across all medical graduates, as mandated by the RCSEng [14]. Foundation doctors frequently provide ward-based wound care, diabetic foot reviews, analgesia prescribing, and referrals to rehabilitation teams [22]. These responsibilities require a baseline understanding of surgical principles, rehabilitation pathways, and psychosocial support before graduation. Amputation care should therefore be regarded as a fundamental component of undergraduate education, rather than a competency deferred to postgraduate training.

Regional analysis revealed significant disparities in both formal teaching exposure and clinical encounters with patients with amputations across UK medical schools. Students in Scotland reported the highest median number of dedicated teaching sessions, while students in Wales reported the lowest median clinical exposure. These findings suggest that undergraduate education in amputation care is not uniformly delivered nationwide, potentially reflecting differences in regional healthcare infrastructure, curricular priorities, or access to specialist services.

Addressing these inconsistencies is essential for equipping future doctors, regardless of training location, with the skills and knowledge required to provide competent, compassionate care to patients undergoing or living with amputation. The absence of structured teaching in this domain misses a valuable opportunity to reinforce broader competencies, including shared decision-making, patient-centred care, and the management of complex disabilities- core competencies expected of all Foundation Year doctors [22].

### Distinguishing aetiologies: why the cause of amputation matters

Although preventive strategies and immediate management priorities may differ depending on the underlying cause of amputation, the post-amputation needs of patients are broadly universal. Regardless of etiology, all patients require surgical wound management, timely access to prosthetic services, multidisciplinary rehabilitation, and psychological support. These shared challenges highlight that amputation care should be considered a holistic competency, relevant to all future doctors. At the same time, understanding etiology-specific factors equips clinicians to anticipate preventive strategies and tailor rehabilitation priorities to individual patients.

In cases of amputations resulting from diabetes, the emphasis should centre on managing underlying medical comorbidities to reduce the progression of peripheral arterial disease and neuropathy, the primary drivers of limb loss in this population [23–25]. In contrast, trauma and oncological related amputations often require early surgical intervention and long-term functional rehabilitation [26, 27].

While trauma is the leading cause of upper limb amputations in the UK [1], in the lower limb sequelae of diabetes is the most prevalent [28]. Individuals diagnosed with diabetes are 15 times more likely to undergo an amputation than the general population [1]. Poorly controlled diabetes can lead to chronic hyperglycaemia leading to reduced sensation and blood perfusion to the lower limb, resulting in skin breakdown, muscle wasting, an increased risk of infection, and delayed wound healing [23, 25, 29]. As a result, individuals with diabetes are prone to foot complications, including ulcer formation. Diabetic foot ulcers precede over 80% of amputations in people with diabetes [28, 30, 31]. Mortality rates are high, with an estimated 70% of individuals dying within five years of an amputation and around 50% within five years of developing a diabetic foot ulcer [28, 32]. The financial burden is substantial, with diabetic foot care alone estimated to cost NHS England £650 million to £1 billion annually [23, 28], exceeding the combined healthcare costs of breast, prostate, and lung cancer [28].

Incorporating dedicated teaching sessions on amputations, particularly in the context of diabetes, is of high relevance, given that an estimated 4.6 million people in the UK have a diabetes diagnosis [33]. Undergraduate curricula should prioritise early prevention strategies, including National Institute for Health and Care Excellence (NICE) guideline-directed care [28], diabetic foot screening, and patient education [24]. Final-year students, soon to be junior doctors, are well positioned to impact patient outcomes through explaining conditions, providing advice and support to patients, formulating treatment and management plans, assisting patients in making decisions about their care (including self-care), and offering information on medications [22]. A 2015 national study reported that at least 75 percent of Foundation Year doctors performed these tasks at least once or twice a week [22].

In contrast, trauma and cancer-related amputations, which are more likely to affect younger patients [34], shift the emphasis from prevention to rehabilitation and functional recovery [26]. Effective rehabilitation helps restore activities of daily living, enabling persons with amputations to return to work and participate in meaningful life roles [35]. The World Health Organisation (WHO) emphasises that rehabilitation allows individuals to be as independent as possible and to reintegrate into education, employment, and family and social life [36]. Trauma-related amputations often require long periods of rehabilitation, and insufficient rehabilitative support can severely impair quality of life [27].

With trauma remaining the leading cause of upper limb amputations in the UK [1] and becoming especially relevant in the context of ongoing global conflicts [5–8, 11, 12]. Students must understand the role of early multidisciplinary input- physiotherapy, prosthetics, psychological support in restoring independence and quality of life.

### The role of charitable and specialist organisations

These findings were shared with Blesma (The Limbless Veterans charity) [17] who noted that the lack of formal amputation teaching was “not surprising” and stressed that early undergraduate exposure to limb loss would benefit both patients and healthcare professionals. They emphasised that well-meaning but uninformed comments about prostheses, rehabilitation expectations, or surgical decisions can have lasting negative impacts. Blesma has called for dedicated sessions within medical school curricula covering the reasons for amputation, the rehabilitation journey, and the social dimensions of limb loss. They advocate that this teaching be developed in partnership with patients, carers, and limb loss charities and formally assessed as part of the medical curriculum.

### Recommendations for future education

Given that 83.3% of students felt their education in this area was inadequate, several changes to undergraduate education should be considered. First, medical curricula should include structured, compulsory teaching on amputation care that spans aetiology, surgery, rehabilitation, and psychosocial support. Within this, diabetic limb preservation and management should be taught as a priority public health issue. Teaching on trauma-related amputations should cover both the surgical indications and long-term functional outcomes, with consideration of global health and conflict perspectives.

Secondly, clinical exposure must be improved. This could involve attachments to rehabilitation units, vascular surgery clinics, or diabetic foot teams. Where in-person exposure is not feasible, simulation, virtual reality, or case-based e-learning could offer accessible alternatives. Thirdly, amputation care should be incorporated into assessments and practical skills teaching—aligning it with its real-world clinical significance. This could include Objective Structured Clinical Examination (OSCE) scenarios, multiple choice questions (MCQ) on diabetic foot management, or reflective assignments involving patient interaction. Finally, medical schools should partner with specialist organisations like Blesma to deliver teaching sessions involving patients with lived experience. These sessions can profoundly shape students’ understanding, empathy, and clinical confidence.

### Strengths and limitations

This study's strengths include it being the first nationwide evaluation of amputation care education across all medical schools in the UK, ethical rigour, and the breadth of data collected, capturing diverse perspectives across UK medical schools. Collaborator recruitment through Plastic, Reconstructive, and Aesthetic Surgery Student Association (PRASSA) ensured consistent survey distribution across all eligible institutions. Additionally, input from Blesma and amputation care specialists during survey development ensured content validity and stakeholder relevance.

However, limitations include the use of a non-random sampling method, which may limit generalisability, and potential response (selection and recall) bias due to the self-reported nature of the survey. Students may have encountered patients with amputations but not recalled these at the time of survey completion. Similarly, if amputation-related content was embedded within other specialty teaching, students may not have recognised or reported this as dedicated amputation education. Additionally, the online format may have disproportionately attracted participants with a pre-existing interest in amputation care. Although the survey reached all UK medical schools, response rates varied, and medical schools with lower engagement may be underrepresented. Having access to local university curriculums to compare student reported exposure to amputation teaching with the university timetables/sessions would have provided a more accurate analysis.

### Future research

Future research could triangulate student-reported exposure with direct curriculum audits, by engaging curriculum heads to confirm whether amputation care learning objectives, as mandated by the RCSEng, are integrated into their programmes. Furthermore, future studies could incorporate core knowledge and concept-based questions into survey instruments to directly assess knowledge gaps alongside perceived confidence levels. This approach would provide stronger validation of the hypothesis that limited teaching and exposure contribute to both reduced confidence and reduced knowledge. Another potential avenue for future work would be the development of a standardised asynchronous online module on amputation care, which could be adopted by medical schools nationally to ensure consistent undergraduate exposure. Such a resource could also be adapted for patients, helping to improve awareness and engagement in the amputation and rehabilitation process. In addition, future studies could benchmark amputation education against similarly prevalent conditions, such as burns, where a recent national collaborative study demonstrated that over 80% of UK students felt their undergraduate teaching was inadequate [16]. Incorporating such comparators would provide valuable context and clarify whether amputation care is uniquely under-represented or part of a broader pattern of neglected surgical education.

## Conclusions

In conclusion, this study highlights significant gaps in undergraduate medical education regarding amputation care, with limited exposure, inadequate teaching, and low confidence levels among final-year medical students. Given the increasing prevalence of amputations in the UK and the critical role of junior doctors in patient management [22], it is essential to integrate more structured and comprehensive teaching strategies into medical curricula. Prioritising education on diabetes-related amputations, trauma-related amputations, and rehabilitation can enhance students' preparedness for clinical practice. Expanding hands-on training through high-fidelity simulations, interprofessional learning, and increased clinical placements will not only improve competency but also ensure that future healthcare professionals can deliver high-quality care to patients with amputations. Addressing these educational deficiencies is vital for optimising patient outcomes and equipping medical graduates with the necessary skills to manage amputation care effectively. Finally, the development of a national undergraduate amputation care curriculum, supported by regulatory bodies and specialist organisations—would ensure consistent, equitable training across all UK medical schools and better equip graduates to deliver holistic, high-quality care.

## Supplementary Information

Supplementary Material 1

Supplementary Material 2

Supplementary Material 3

Supplementary Material 4

## Data Availability

The datasets generated and analysed during the current study are available from the corresponding author (Ameen Mahmood) upon reasonable request. The full survey instrument is included as Additional file 1. Supplementary statistical tables are included as Additional file 2 (regional medians), Additional file 3 (teaching exposure comparison), and Additional file 4 (clinical exposure comparison).

## Abbreviations

ANOVA: Analysis of Variance
GDPR: General Data Protection Regulation
GMC: General Medical Council
IQR: Interquartile Range
IWGDF: International Working Group on the Diabetic Foot
MCQ: Multiple Choice Question
MDT: Multidisciplinary Team
NHS: National Health Service
NICE: National Institute for Health and Care Excellence
OSCE: Objective Structured Clinical Examination
PRASSA: Plastic, Reconstructive, and Aesthetic Surgery Student Association
RCSEng: Royal College of Surgeons of England
SD: Standard Deviation
SSCs: Student Selected Components
UK: United Kingdom
WHO: World Health Organisation
