# Supplementary material for: Undergraduate exposure and confidence to amputations and amputee care: a national survey of final-year UK medical students
Source: BMC Med Educ. 2025 Nov 27;25:1658. doi: 10.1186/s12909-025-08027-4 (PMC12659368; doi:10.1186/s12909-025-08027-4)
Supplement: Supplementary file 1 — Supplementary Material 1 [file 12909_2025_8027_MOESM1_ESM.docx]

## Section 1: Demographics

1. What is your medical school? (Dropdown list of all UK medical schools)

2. Are you in final year of medical school? (Yes/No)

## Section 2: Exposure to Amputation Teaching

3. How many dedicated teaching sessions (e.g., lectures, tutorials, seminars) have you received specifically on amputations and/or amputation care during your medical education thus far? (None, 1 / 2–3 / 4–5 / 6–7 / >7)

4. Which formats of teaching on amputations and/or amputation care have you experienced? (Select all that apply):

   - Lectures
   - Tutorials/Seminars
   - Clinical Skills Sessions
   - Simulation-based Training
   - Clinical Observations of patients with amputations
   - Formal clinical rotation or placement that included exposure to amputations and/or amputation care
   - None
   - Other

5. How effective do you think the teaching sessions you’ve attended on amputations and/or amputation care have prepared you to provide care for patients with amputations? (Likert Scale: Very ineffective- Very effective and Not Applicable)

6. Approximately how many patients with amputations have you encountered or interacted with during your medical training thus far? (None / 1–5 / 6–10 / 11–15 / 16-20 / >20)

7. How would you rate the adequacy of clinical exposure to patients with amputations during your undergraduate medical training? (Likert Scale: Very Inadequate- More than Adequate)

8. Have you observed an amputation operation during your undergraduate medical training? (Yes/No)

9. Have you had hands-on experience with procedures directly related to amputation care, such as wound dressing, stump care, or prosthetic fitting, during your undergraduate medical training? (Yes/No)

10. What do you perceive as the primary barriers to effective learning and understanding of amputations and/or amputation care during your medical education? (Select all that apply)

- Limited dedicated teaching sessions
   - Lack of clinical exposure
   - Inadequate resources/materials
   - Time constraints in the curriculum
   - Clinical Observations of patients with amputations
   - Lack of emphasis on amputations and/or amputation care in assessments
   - Other

## Section 2: Student Confidence Levels

Please indicate your agreement with the following statements (Likert scale: Strongly disagree – Strongly agree):

11. I feel confident in my knowledge of the psychological effects that amputations can have on patients.

12. I feel confident in my knowledge of the impact that amputations can have on a patient's overall physiological health.

13. I feel confident in my knowledge of the resources and services available to support patients with amputations (e.g., rehabilitation, prosthetics, support groups).

14. How much do you agree with the following statement: "I am aware of the various specialists (medical/surgical and allied professionals) involved in the multidisciplinary team for amputation care"?

## Section 4: Perceptions and Attitudes Towards Amputation

15. How do you perceive individuals with amputations? (Likert scale: Very negative – Very positive)

16. How significant do you perceive amputations to be compared to other major surgical procedures (e.g., cardiac surgery, organ transplantation)? (Likert scale: Much less significant – Much more significant)

## Section 5: Feedback on Amputation Care Teaching

17. How do you perceive your overall competence in providing care for patients with amputations upon graduation from medical school? (Likert scale: Not confident at all – Very confident)

18. How much do you agree with the following statement: "I have received adequate teaching in amputations and/or amputation care during my undergraduate medical education thus far?" (Likert scale: Strongly Disagree – Strongly Agree)

19. How much do you agree with the following statement: "Greater exposure and teaching are required on amputations and/or amputation care at the undergraduate level"? (Likert scale: Strongly Disagree – Strongly Agree)

20. Would you attend a teaching session or workshop related to amputations and amputation care? (Yes/No)
