# Supplementary material for: Undergraduate exposure and confidence to amputations and amputee care: a national survey of final-year UK medical students
Source: BMC Med Educ. 2025 Nov 27;25:1658. doi: 10.1186/s12909-025-08027-4 (PMC12659368; doi:10.1186/s12909-025-08027-4)
Supplement: Supplementary file 2 — Supplementary Material 2 [file 12909_2025_8027_MOESM2_ESM.docx]

**Table 1.** Regional comparison of median number of dedicated amputation teaching sessions received and median number of amputee patients encountered during training, summarised with interquartile ranges (IQR). Regions are grouped according to UK medical school affiliations. Significant differences between regions were observed for both teaching sessions (p < 0.001) and clinical exposure (p < 0.001) based on Kruskal-Wallis tests.

| Region | Teaching sessions  (median ± IQR) | Patients encountered  (median ± IQR) |
| --- | --- | --- |
| National | 0.00 ± 1.00 | 3.00 ± 5.00 |
| Scotland | 1.00 ± 2.50 | 3.00 ± 5.00 |
| Northern Ireland | 0.00 ± 0.50 | 3.00 ± 2.50 |
| North East & Cumbria | 0.00 ± 1.00 | 3.00 ± 5.00 |
| Yorkshire & Humber | 0.00 ± 2.50 | 3.00 ± 3.75 |
| Northwest | 0.00 ± 1.00 | 3.00 ± 5.00 |
| West Midlands | 0.00 ± 0.00 | 3.00 ± 5.00 |
| East Midlands | 0.00 ± 1.00 | 3.00 ± 0.00 |
| Wales | 0.00 ± 0.00 | 0.00 ± 3.00 |
| East of England | 0.00 ± 0.50 | 3.00 ± 2.50 |
| London | 0.00 ± 0.50 | 3.00 ± 0.00 |
| South East | 0.00 ± 0.00 | 3.00 ± 0.00 |
| South West | 0.00 ± 0.00 | 3.00 ± 0.00 |
