# Supplementary material for: Undergraduate exposure and confidence to amputations and amputee care: a national survey of final-year UK medical students
Source: BMC Med Educ. 2025 Nov 27;25:1658. doi: 10.1186/s12909-025-08027-4 (PMC12659368; doi:10.1186/s12909-025-08027-4)
Supplement: Supplementary file 3 — Supplementary Material 3 [file 12909_2025_8027_MOESM3_ESM.docx]

**Table 1.** Comparison of median ± interquartile range (IQR) of Likert scores from national data on confidence, competence, and perceptions regarding amputation care between students with and without teaching sessions based on Mann-Whitney U tests.

Note: The p-values indicate the statistical significance of differences between students who attended at least 1 dedicated teaching session on amputation care and those with no teaching sessions.

|  | National Data | Teaching Sessions versus No Teaching Sessions | | |
| --- | --- | --- | --- | --- |
|  | National average  (median ±  IQR) | Teaching Sessions  (median ±  IQR) | No Teaching Sessions  (median ±  IQR) | p value |
| I feel confident in my knowledge of the psychological effects that amputations can have on patients. | 3.00 ± 2.00 | 3.00 ± 1.00 | 2.00 ± 1.00 | <0.001 |
| I feel confident in my knowledge of the impact that amputations can have on a patient's overall physiological health. | 2.00 ± 1.00 | 3.00 ± 2.00 | 2.00 ± 1.00 | <0.001 |
| I feel confident in my knowledge of the resources and services available to support patients with amputations (e.g., rehabilitation, prosthetics, support groups). | 2.00 ± 2.00 | 2.00 ± 1.00 | 2.00 ± 1.00 | <0.001 |
| How much do you agree with the following statement: "I am aware of the various specialists (medical/surgical and allied professionals) involved in the multidisciplinary team for amputation care"? | 2.00 ± 2.00 | 3.00 ± 2.00 | 2.00 ± 1.00 | <0.001 |
| How do you perceive individuals with amputations? | 3.00 ± 1.00 | 3.00 ± 2.00 | 3.00 ± 1.00 | 0.019 |
| How significant do you perceive amputations to be compared to other major surgical procedures (e.g., cardiac surgery, organ transplantation)? | 3.00 ± 1.00 | 3.00 ± 1.00 | 3.00 ± 1.00 | 0.728 |
| How do you perceive your overall competence in providing care for patients with amputations upon graduation from medical school? | 2.00 ± 2.00 | 2.00 ± 1.00 | 2.00 ± 1.00 | <0.001 |
| How much do you agree with the following statement: "I have received adequate teaching in amputations and/or amputation care during my undergraduate medical education thus far?" | 2.00 ± 1.00 | 2.00 ± 1.00 | 2.00 ± 1.00 | <0.001 |
| How much do you agree with the following statement: "Greater exposure and teaching are required on amputations and/or amputation care at the undergraduate level"? | 4.00 ± 1.00 | 4.00 ± 1.00 | 4.00 ± 1.00 | 0.658 |
